# Combination of AURKA inhibitor and MEK inhibitor strongly enhances G1 arrest and induces synergistic antitumor effect on *KRAS* or *BRAF* mutant colon cancer cells

**DOI:** 10.1016/j.bbrep.2025.102073

**Published:** 2025-06-09

**Authors:** Masashi Sato, Yoshiyuki Yamamoto, Toshikazu Moriwaki, Kuniaki Fukuda, Kiichiro Tsuchiya

**Affiliations:** a Department of Gastroenterology, Institute of Clinical Medicine, Graduate School of Comprehensive Human Sciences, University of Tsukuba, 1-1-1 Tennodai, Tsukuba, Ibaraki, 305-8575, Japan; b Department of Gastroenterology and Hepatology, Kurashiki Central Hospital, 1-1-1 Miwa, Kurashiki, Okayama, 710-8602, Japan

**Keywords:** AURKA, MEK, Colon cancer, ERK, Cell cycle, Apoptosis

## Abstract

In colorectal cancer, *RAS* and *BRAF* are major mutation points in the RAS-MAPK signaling pathway. These gene mutations are known to be important causes of resistance to anti-EGFR antibody therapies. Recently, it has been reported that Aurora kinase A (AURKA), one of the mitotic kinases, interacts with the EGFR-RAS-MAPK signaling pathway. In this study, we examined whether the combination of MK-5108 (AURKA inhibitor) and trametinib (MEK inhibitor) enhanced the antitumor effect for colon cancer cell lines. The combination of MK-5108 and trametinib showed synergistic enhancements of antitumor effect in three colon cancer cell lines harboring *KRAS* or *BRAF* mutation. Cell cycle analysis showed induction of G2/M and G1 arrests by MK-5108 and trametinib, respectively, and the potential enhancement of G1 arrest with the two drug combination. The addition of MK-5108 to trametinib enhanced the suppression of *p*-ERK and other G1/S progression-related proteins expression. In HCT116 cells, harboring wild-type *TP53*, the combination therapy induced more potent cell proliferation suppression and apoptosis induction than in *TP53* knockout cells. These were related to potential enhancement of p53 expression and caspase activation. In conclusion, the combination of MK-5108 and trametinib may synergistically inhibit tumor cell division with or without *TP53* mutation, and with either *KRAS* or *BRAF* mutation. Furthermore, the combination therapy could be more effective in wild-type *TP53* cells.

## Highlights

• The combination of MK-5108 and trametinib induced G1 arrest in colon cancer cells.
• The addition of MK-5108 to trametinib enhanced the suppression of *p*-ERK expression.
• In wild-type *TP53* cells, trametinib enhanced apoptosis promotion effect of MK-5108.
• Effects of MK-5108 and trametinib are enhanced regardless of *TP53*, *RAS*, *BRAF* status.

## 1 Introduction

Anti-epidermal growth factor receptor (EGFR) antibodies are now frequently used as one of the standard therapies for colorectal cancer. However, activating mutations in the RAS-MAPK signaling pathway, represented by *RAS* and *RAF*, are important causes of resistance to anti-EGFR antibody therapies. Although targeted therapies for a few types of *RAS/RAF* mutant colorectal cancers (such as those with *KRAS*^*G12C*^ and *BRAF*^*V600E*^ mutations) have been clinically applied in recent years [1], these mutations are relatively rare, and no effective treatment has been developed for most patients with other *RAS/BRAF* mutations.

MEK is a downstream molecule of the RAS-MAPK signaling pathway and was hoped to be a new target of treatment for *RAS/RAF* mutant tumors. Many MEK inhibitors have been developed, and some have progressed to clinical trials for several tumors, including colon cancer. Regrettably, the antitumor effect of MEK inhibitors is not sufficient as monotherapy because reactivation of RAS-MAPK signaling is caused by a feedback mechanism and reduces the MEK inhibition effect. It is thought that the RAS-MAPK signaling pathway should be suppressed at multiple points to enhance the antitumor effect of MEK inhibitors. Currently, MEK inhibitors are used clinically in combination with other drugs for tumors with specific genetic mutations [2].

Aurora kinase A (AURKA), one of the mitotic kinases, is known to play an important role in the late G2 phase to the early M phase of cell division, including centrosome maturation, spindle assembly, entry to mitosis, and cytokinesis [3]. Gene amplification and overexpression of AURKA have been observed in various cancers, and a relationship between AURKA overexpression and poor prognosis has also been reported, suggesting that AURKA may function as an oncogenic driver [3]. Inhibition of AURKA activity induces suppression of cell proliferation, and many AURKA inhibitors have been developed. MK-5108 is a highly selective small molecular inhibitor of AURKA. MK-5108 induces G2/M arrest and apoptosis and has shown antitumor effects both in vitro and in vivo [4]. Recently, several studies have pointed out that AURKA interacts with the EGFR-RAS-MAPK signaling pathway and that they upregulate each other [[5], [6], [7]]. Thus, we hypothesized that the addition of an AURKA inhibitor to a MEK inhibitor may serve to enhance suppression of the RAS-MAPK signaling pathway. In the present study, we investigated whether a combination of the AURKA inhibitor MK-5108 and the MEK inhibitor trametinib shows a synergistic antitumor effect in colon cancer cell lines harboring several different phenotypes (*TP53* wild/mutant and *KRAS* or *BRAF* mutant). Furthermore, we explored the molecular mechanisms of the antitumor effect of the combination therapy both in terms of cell cycle arrest and apoptosis.

## 2 Materials and methods

### 2.1 Reagents

The selective aurora kinase A inhibitor MK-5108 was purchased from Chemscene (Monmouth Junction, NJ, US), and the MEK inhibitor trametinib was purchased from Cayman Chemical (Ann Arbor, MI, US). MK-5108 and trametinib were dissolved in DMSO to concentrations of 10 mmol/L and 324 μmol/L, respectively.

### 2.2 Cell lines and culture

Three human colon cancer cell lines (HCT116, DLD1, HT29) and one isogenic variant (HCT116p53−/−) were used. HCT116 and HCT116p53−/− were obtained from Horizon Discovery (Cambridge, UK). DLD1, and HT29 were obtained from ATCC (Rockville, MD, US). HCT116, HCT116p53−/−, and DLD1 cells were cultured in RPMI 1640 medium (MilliporeSigma, Burlington, MA, US) supplemented with 10 % fetal bovine serum (FBS) (MilliporeSigma). HT29 cells were cultured in McCoy's 5 A medium (Thermo Fisher Scientific, Waltham, MA, US) with 10 % FBS.

### 2.3 Cell viability analysis and combination index

Cell viabilities were measured by WST-8 assay with Cell Count Reagent SF (Nacalai Tesque) as previously described [8]. Cells were cultured overnight. MK-5108 and trametinib were added alone or in combination and cultured for 3 days. The efficacy of the combination therapy was evaluated using the Chou-Talalay method for drug combination using CalcuSyn software (Biosoft, Cambridge, UK) [9]. A combination index (CI) < 0.9 indicates synergism, CI = 0.9–1.1 indicates additivity, and CI > 1.1 indicates antagonism.

### 2.4 Cell cycle analysis

Cells were cultured overnight, agents were added, and then cultured for another 22 h. EdU was then added to the medium and cultured for another 2 h. EdU assay was performed for harvested cells using Click-iT Plus EdU Alexa Fluor 647 Flow Cytometry Assay Kit (Thermo Fisher) according to the manufacturer's protocol. Flow cytometry was performed using BD FACSVerse, and the percentage of each cell cycle phase was calculated using FlowJo software (both produced by BD Biosciences, Franklin Lakes, NJ, US).

### 2.5 Cell death analysis

After adding the agents, cells were cultured for 24 h and then harvested. Cells were stained with propidium iodide using Cycletest Plus DNA reagent kit (BD Biosciences). Flow cytometry was performed using BD FACSVerse, and the sub-G1 fraction was analyzed using FlowJo.

### 2.6 Immunoblot analysis

Both SDS-PAGE and immunoblot analysis were performed as previously described [8]. The primary and secondary antibodies used in this study are shown in Table S1. Chemiluminescent reactions were visualized by ECL Select Western Blotting Detection Reagent (GE Healthcare), and Ez-Capture Imaging System (Atto, Tokyo, JP).

### 2.7 Statistical analysis

Data are presented as mean ± standard deviation (SD). Analysis of statistical significance between three or more groups was performed using one-way ANOVA with Tukey's test for post hoc analysis. If equality of variance was not shown by Levine's test, the significance was evaluated using the Games-Howell test. P values < 0.05 were considered statistically significant. Statistical analysis was carried out using SPSS version 26.0 (IBM, Armonk, NY, US).

## 3 Results

### 3.1 Combination of MK-5108 and trametinib shows a synergistic antitumor effect

**Fig. 1:**
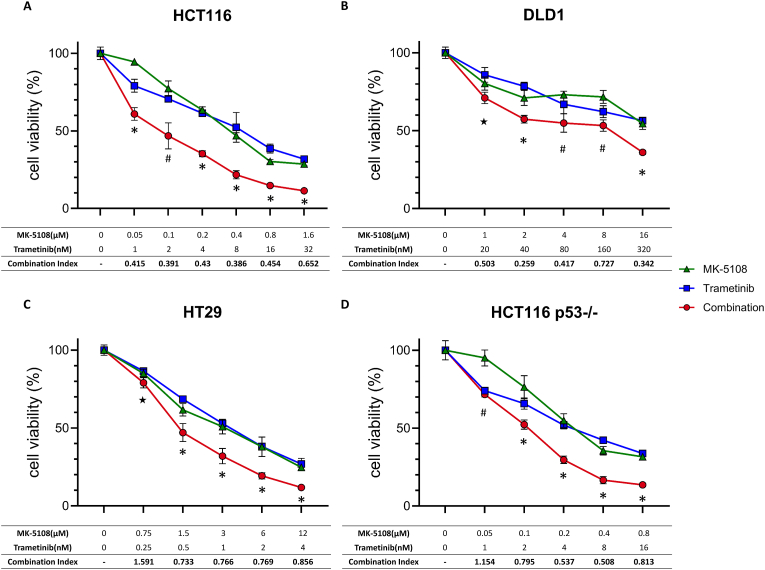
Antitumor effects of MK-5108-alone and trametinib-alone or in combination in colon cancer cell lines. Cells were exposed to different dose levels of MK-5108 and/or trametinib for 3 days. Control was exposed to DMSO. Cell viabilities were measured using WST-8 assay (mean ± SD; n = 3). Statistical significance between combination and MK-5108 or trametinib was evaluated. ∗P-value <0.05 Combination vs. both MK-5108 and trametinib. ^#^P-value <0.05 Combination vs. MK-5108. ^★^P-value <0.05 Combination vs. Trametinib. CIs were calculated at each concentration.

To investigate the efficacy of the combination of MK-5108 and trametinib, we examined the antitumor effect using WST-8 assay in colon cancer cell lines: HCT116 cells harboring wild-type (wt) *TP53* and mutant-type (mt) *KRAS*, DLD1 harboring mt *TP53* and mt *KRAS*, and HT29 harboring mt *TP53* and mt *BRAF* (Fig. 1A–C). Monotherapy of MK-5108 or trametinib reduced cell viabilities of these cell lines in a dose-dependent manner, and combination therapy resulted in an even greater reduction. CIs of these cell lines were below 0.9 except for the lowest dose level of HT29, indicating that the combination therapy synergistically enhances the antitumor effect. In all cell lines, CIs were relatively lower during the combination therapy with nearly 50 % of the suppression dose levels of MK-5108 and trametinib (The IC_50_ values for each cell line are shown in Table S2.). Therefore, we set the concentrations of MK-5108 and trametinib as follows: HCT116 (0.4 μM, 8 nM), DLD1 (16 μM, 320 nM), and HT29 (3 μM, 1 nM).

### 3.2 The potential effect of combination therapy on G1 phase arrest induction and inhibition of cell cycle progression

**Fig. 2:**
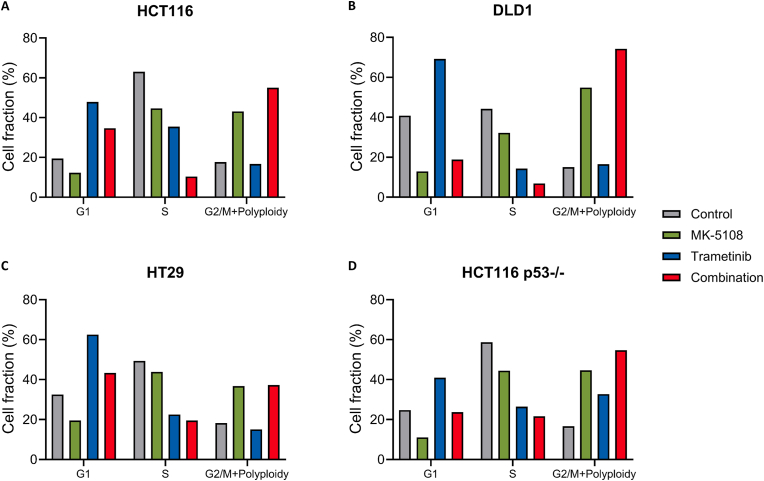
Effects of MK-5108 and trametinib on cell cycle. Cells were exposed to MK-5108 and/or trametinib for 24 h. Control was exposed to DMSO. Cells were analyzed by flow cytometry using EdU assay. The S phase fraction was determined as EdU-positive cells.

Since both MK-5108 and trametinib induce cell cycle arrest at different phases, we examined the influence of the combination therapy on the cell cycle in HCT116, HT29, and DLD1 cells. In all cell lines, as previously suggested [10,11], MK-5108 monotherapy increased G2/M phase and polyploid cell fraction, trametinib monotherapy increased G1 phase cell fraction, and both decreased the S phase cell fraction (Fig. 2A–C and S1A-C). When these drugs were combined, more pronounced reduction of S phase cells were found, suggesting an enhancement of cell cycle arrest. We also evaluated the number of cell divisions of HCT116 cells under MK-5108, trametinib, or the combination therapy. As shown in Fig. S2, the division index with the combination therapy was less than those with monotherapies (Fig. S2). This result shows that MK-5108 and trametinib do indeed inhibit cell division, and that the combination therapy inhibits it more potently. Although the G2/M phase and polyploid cells were increased with the combination therapy, proportion of positive cells for phospho-Histone H3 (p-HH3), known as a mitosis marker, was lower compared to the MK-5108 monotherapy (Fig. S3). It may suggest that the observed increase of G2/M phase and polyploid fraction was not indicative of increased cells arrested in M phase. Mitotic arrest is known to result in three types of cell fates: complete cell division, cell death, or mitotic slippage which leads to polyploidy [12]. Our findings possibly suggest that the combination therapy mainly induces polyploid cells which were expected to be in non-S, non-M phase. Thus, we expect that cell cycle arrest, and especially the G1 phase arrest, plays an important role in enhancing the antitumor effects of the combination of MK-5108 and trametinib.

### 3.3 The combination therapy may inhibit expressions of G1/S progression-related proteins

**Fig. 3:**
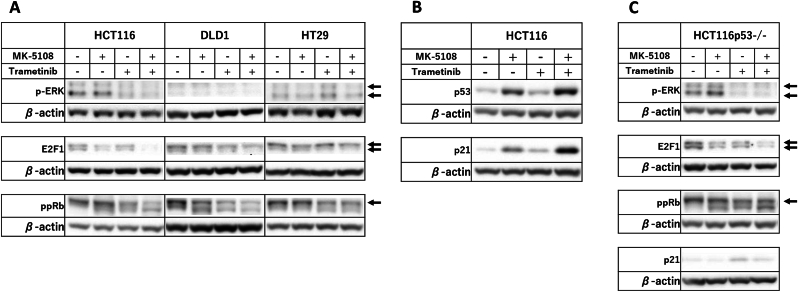
Expression of *p*-ERK and G1/S progression-related proteins in cell lines. Cells were exposed to drugs for 24 h and then analyzed for *p*-ERK, E2F1, hyperphosphorylated Rb (ppRb), p53, and p21 expression levels by immunoblot analysis. β-actin was used as an internal control.

To explore the molecular mechanism of G1 arrest promoted by the combination therapy, we examined G1/S progression-related proteins using immunoblot analysis (Fig. 3A). In all cell lines, the combination therapy appeared to reduce expressions of *p*-ERK, a downstream factor of MEK and an important factor for cell cycle, especially G1/S progression. pp-Rb and E2F1, two other important factors of G1/S progression, also tended to decrease more with the combination therapy compared to the monotherapies, suggesting a possible association of the synergistic suppression of ERK activity and other downstream factors to the observed robust cell cycle arrest.

With HCT116 cells which harboring wt *TP53*, we sought to explore expression of CDK inhibitor p21, which suppresses G1/S progression, and p53, a transcription factor of p21 to examine the combination therapy effect. Immunoblotting potentially suggested enhanced expressions of both p21 and p53 by MK-5108 which were further increased with addition of trametinib (Fig. 3B). As we hypothesized that an activation of p53 may contribute to acceleration of cell cycle arrest in wt *TP53* tumor cells, we additionally analyzed the cell cycle and expression of cell cycle-related proteins in HCT116p53−/− cells. As shown in Fig. 2D and S1D, impact of the combination therapy on S phase cells reduction in HCT116p53−/− cells was limited compared to that of HCT116 cells. Although the number of cell divisions was least with the combination therapy in HCT116p53−/− cells, the alteration of division index of HCT116p53−/− cells by combination therapy remained 64 % while that of HCT116 cells reached 74 % (Fig. S2 and S4). Further, immunoblotting showed that these cells potentially harbor similar amount of p21 and pp-Rb with or without the combination therapy whereas *p*-ERK expression was appeared to be strongly suppressed (Fig. 3C). These results suggest that the combination therapy may induce cell cycle arrest more strongly in wt *TP53* cells.

### 3.4 Apoptosis is promoted with the combination therapy in wt TP53 cells

**Fig. 4:**
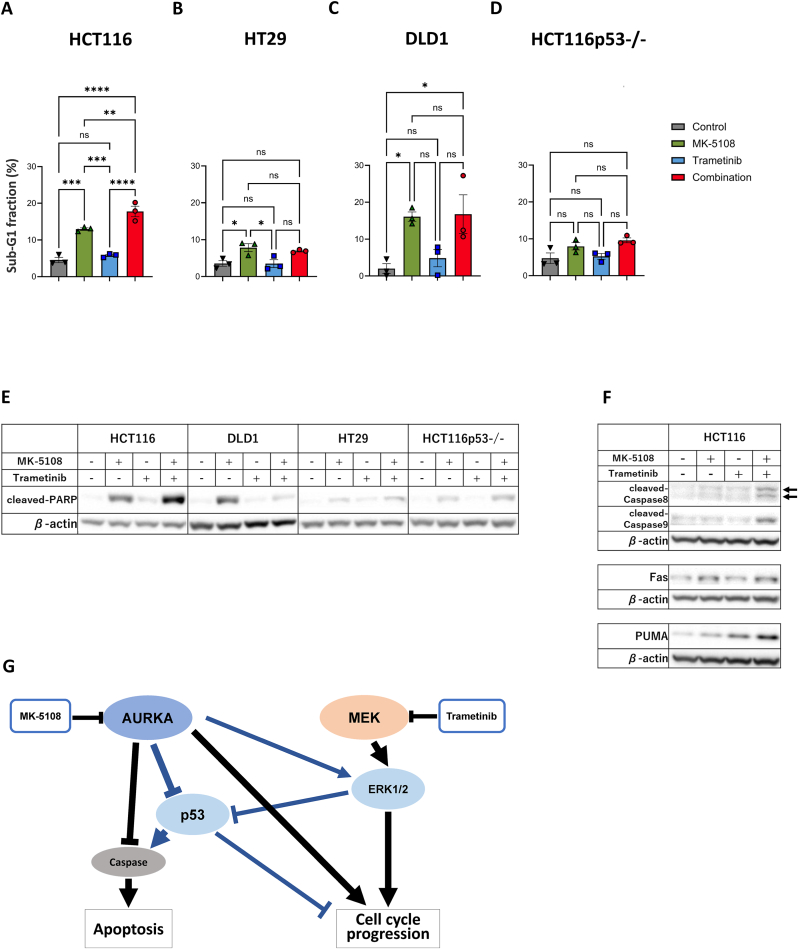
Apoptosis induction effects of MK-5108 and trametinib in cell lines. **A-D** Increase in the sub-G1 fraction. Cells were exposed to drugs for 24 h, and sub-G1 (<2 N) fractions were evaluated by flow cytometry using propidium iodide (mean ± SD; n = 3). Statistical significance between groups was evaluated. ∗P-value <0.05, ∗∗P-value <0.01, ∗∗∗P-value <0.001, and ∗∗∗∗P-value <0.0001. **E, F** Expression of apoptosis-related proteins in cell lines. Cells were exposed to drugs for 24 h and then analyzed for cleaved-PARP, cleaved-Caspase 8, cleaved-Caspase 9, Fas, and PUMA expression levels by immunoblot analysis. β-actin was used as an internal control. **G** Schematic mechanism of the antitumor effect of the combination of MK-5108 and trametinib. MK-5108 decreases AURKA activity and induces G2/M arrest and apoptosis. Trametinib decreases ERK activity and induces G1 arrest. AURKA inhibition also reduces the RAS-MAPK signal. Synergistic inhibition of ERK activity by MK-5108 and trametinib induces potent G1 arrest. Furthermore, in wild-type *TP53* cells, p53 is upregulated by both AURKA inhibition and ERK inhibition. Overexpressed p53 promotes further G1 arrest and caspase-mediated apoptosis.

Next, we analyzed the effect of the combination of MK-5108 and trametinib on the induction of cell death (Fig. 4A and S5A). In HCT116 cells, MK-5108 monotherapy increased sub-G1 fraction, known to reflect apoptosis, while trametinib monotherapy did not show a clear increase. However, the addition of trametinib to MK-5108 increased the sub-G1 fraction more than the MK-5108 monotherapy. LDH assay showed that the combination therapy increased the number of dead cells relative to viable cells compared to MK-5108 or trametinib monotherapy (Fig. S6). These results suggest that the combination therapy also promoted cell death more effectively than monotherapy in HCT116 cells. However, the same results were not obtained in mt *TP53* cell lines including HCT116p53−/− (Fig. 4B–D and S5B-D). The MK-5108 monotherapy increased the sub-G1 fraction in all cell lines, but trametinib could not enhance the sub-G1 increasing effect of MK-5108. Next, we examined the expression of cleaved-PARP, known as a marker of apoptosis, in each cell line by immunoblot analysis (Fig. 4E). In HCT116 cells harboring wt *TP53*, the cleaved-PARP expression level appeared to be increased in the order of the combination, MK-5108-alone, and trametinib-alone, and it was correlated with the change of the sub-G1 fraction. On the other hand, DLD1 cells did not show an increase of cleaved-PARP expression with the combination therapy, and HT29 cells showed only a small amount of cleaved-PARP expression. In HCT116p53−/− cells, as with the sub-G1 fraction, cleaved-PARP expression also appeared to be markedly lower compared with its expression in HCT116 cells. The LDH assay showed that cell death was actually reduced in HCT116p53−/− (Fig. S7). Apoptosis-related proteins were examined in HCT116 cells, in which cell death was promoted by the combination therapy (Fig. 4F). Both cleaved-Caspase 8 and cleaved-Caspase 9 (apoptosis activation markers of extrinsic and intrinsic pathways) expressions appeared to be upregulated by the combination therapy. These results suggest that activated p53 potentially promotes apoptosis with the combination therapy in the wt *TP53* cell lines, which may contribute to the synergistic enhancement of the antitumor effect. As shown in Fig. 1D, the antitumor effect of the combination of MK-5108 and trametinib was also investigated in HCT116p53−/− cells. The CIs were below 0.9 at most concentrations, indicating a synergistic enhancement of antitumor effect. However, the CIs were slightly higher than those observed in HCT116 cells.

## 4 Discussion

In this study, we showed that the combination of MK-5108 and trametinib has a synergistic antitumor effect on colon cancer cell lines. Previously, two basic studies about effectiveness of the combination of AURKA inhibitor and MEK inhibitor have been reported, one on melanoma cell lines [13] and the other on colorectal cancer cell lines [14]. However, these studies have not described the involved mechanisms in detail. The main contributions of our study are showing that the combination of AURKA inhibitor and MEK inhibitor is effective regardless of *TP53* mutation and *RAS* or *BRAF* mutation, and revealing in detail potential mechanisms on efficacy of the combination therapy.

We showed that efficient induction of cell cycle arrest was the most important mechanism in the synergistic enhancement of antitumor effects of the combination therapy. In addition to G1 arrest effect of trametinib and G2/M arrest effect of MK-5108, the combination therapy was further associated to G1 arrest and promoted cell cycle arrest. We focused on ERK activity, an important factor of cell cycle progression, as a mechanism of G1 arrest promoted by the combination of MK-5108 and trametinib. ERK is a downstream factor of the RAS-MAPK signaling pathway; however, the addition of the AURKA inhibitor further decreased ERK activity in this study. The relationships between AURKA and the RAS-MAPK signaling pathway have been reported. Although the detailed mechanisms have not yet been revealed, it is thought that AURKA upregulates the RAS-MAPK signaling pathway [7,15,16]. Some previous studies have reported that AURKA inhibition by a small molecular inhibitor [5] or small interference RNA [15] decreased ERK activity in *RAS* mutant cell lines. In this study, MK-5108 enhanced the effect of trametinib on ERK activity reduction, which could be an important factor in the enhancement of G1 arrest.

Furthermore, we showed that wild-type p53 may contribute to the induction of cell cycle arrest. In wt *TP53* cell line, HCT116, MK-5108 increased the expression of p53 and its transcriptional target p21. The relationship between AURKA and p53 has been studied extensively, and it is known that these two molecules form a negative feedback loop. AURKA phosphorylates p53, therefore inhibits the transcriptional activity of p53 and promotes an MDM2-mediated p53 degradation by ubiquitination [17,18]. Our finding was in line with previous reports that showed suppressing AURKA activity increased expression of p53 [19,20]. In this study, the addition of trametinib to MK-5108 in HCT116 cells further enhanced the expression of p53 and p21. Several studies showed that ERK also suppresses p53 via an upregulation of MDM2 [21,22]. The combination of MK-5108 and trametinib strongly induced p53 expression, and it may coordinately promote G1 arrest with decreased ERK activity.

In all cell lines, MK-5108 increased the sub-G1 fraction and may increase expression of cleaved-PARP, indicating apoptosis induction. When mitotic arrest is prolonged in tumor cells by anti-mitotic drugs (such as microtubule targeting agents and mitotic kinase inhibitors), while some cells complete normal division or transition to polyploid cells through mitotic slippage, other cells fail to escape mitotic arrest leading to cell death [12,23]. AURKA also inhibits apoptosis via suppression of FADD [24], induction of Bcl-2 [24,25], and activation of NF-κB [26]. This indicates that AURKA inhibition promotes acceleration and derepression of apoptosis. In this study, while trametinib did not enhance the apoptosis promotion effect of MK-5108 in mt *TP53* cells, the combination therapy enhanced it in wt *TP53* cells, HCT116. Conversely, knockout of p53 seemed to decrease cleaved-PARP expression. These results indicate that p53 plays an important role in apoptosis. In HCT116 cells, we found potential link between the expressions of p53, Fas, cleaved-Caspase 8, and cleaved-PARP. This may suggest that the combination of MK-5108 and trametinib activates the extrinsic apoptosis pathway via p53 activation, inducing apoptosis synergistically.

This study has a few limitations that need to be addressed. First, direct evidence on the G1 phase arrest induction is lacking. It is possible that the combination therapy induced G2 phase arrest in which cells would not proceed to histone H3 phosphorylation. Although increased G1 phase cells in HCT116 and HT29 cells by the combination therapy is suggestive of G1 cell arrest, further studies are required to provide the evidence. Second, the immunoblot experiments were only performed with a small sample number and were not quantitative. Despite all the results were consistent with each other and alterations by the combination therapy were clear, the interpretation of the results needs a caution.

In this study, we demonstrated that the combination of MK-5108 and trametinib shows a synergistic antitumor effect in *RAS/RAF* mutant colon cancer and that the combination therapy may be more effective in wt *TP53* cells. G1 arrest might have play a major role, while apoptosis also contributed in wt *TP53* cells (Fig. 4G). The combination of AURKA inhibitor and MEK inhibitor could be a novel strategy for *RAS/RAF* mutant colon cancer. Additionally, in *RAS/RAF* wild colon cancer, it has been reported that some tumors acquiring resistance to anti-EGFR antibody therapy harbor new mutations in the RAS-MAPK signaling pathway [27]. The combination therapy may constitute salvage therapy for such patients. However, this study was carried out using only in vitro experiments, and further in vivo evaluations are needed, including a comparison of the presence of the *TP53* mutation.

## 5 Conclusion

In conclusion, the combination therapy of an AURKA inhibitor (MK-5108) and a MEK inhibitor (trametinib) exerts a synergistic antitumor effect on colon cancer cells regardless of *TP53* and *RAS/RAF* mutation status via suppression of cell division by promotion of cell cycle arrest. The presence of wt *TP53* may more effectively enhance the antitumor effect through strong cell cycle arrest induction and through synergistic promotion of apoptosis.

## CRediT authorship contribution statement

**Masashi Sato:** Writing – original draft, Investigation, Formal analysis, Conceptualization. **Yoshiyuki Yamamoto:** Writing – review & editing, Supervision, Project administration, Conceptualization. **Toshikazu Moriwaki:** Writing – review & editing, Funding acquisition. **Kuniaki Fukuda:** Writing – review & editing. **Kiichiro Tsuchiya:** Writing – review & editing, Supervision, Conceptualization.

## Availability of data and materials

All data generated or analyzed during this study are included in this published article and its supplementary information files.

## Funding

This work was supported by the scholarship donation from the Pharmaceutical Business Division of Yakult Honsha Co.

## Declaration of competing interest

The authors declare that they have no known competing financial interests or personal relationships that could have appeared to influence the work reported in this paper.
