# Supplementary material for: Combination of AURKA inhibitor and MEK inhibitor strongly enhances G1 arrest and induces synergistic antitumor effect on KRAS or BRAF mutant colon cancer cells
Source: Biochem Biophys Rep. 2025 Jun 9;43:102073. doi: 10.1016/j.bbrep.2025.102073 (PMC12180963; doi:10.1016/j.bbrep.2025.102073)

p-ERK for Figure 3A, 3C

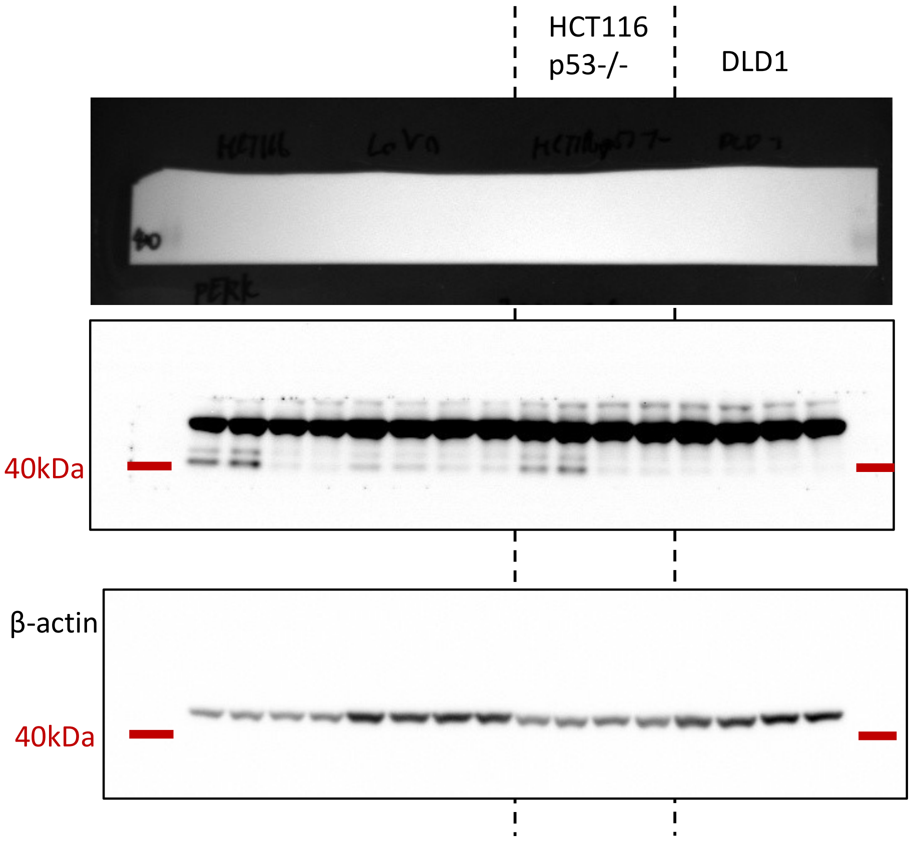

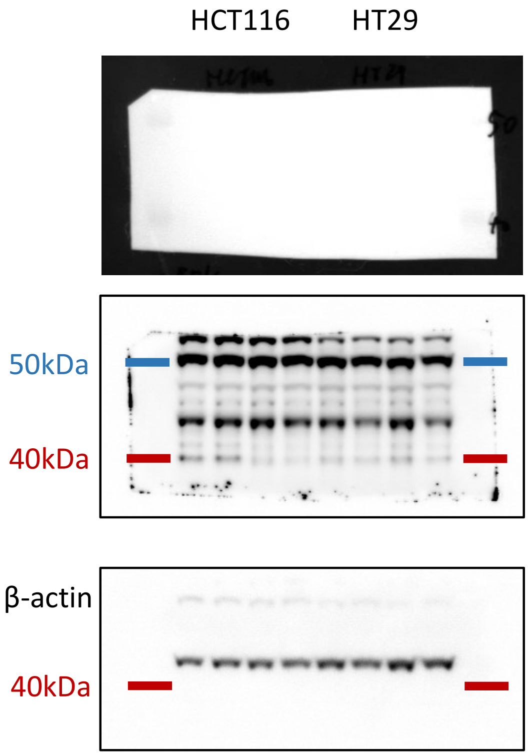

E2F1 for Figure 3A, 3C

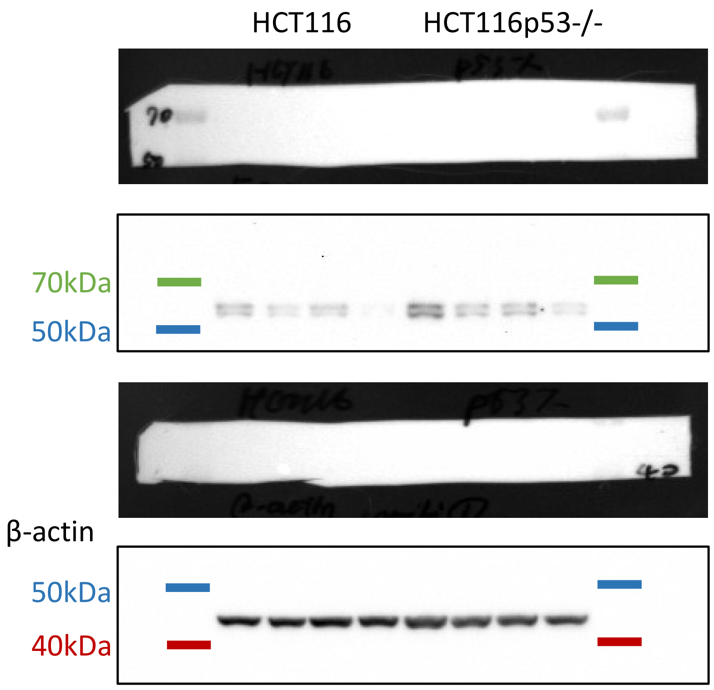

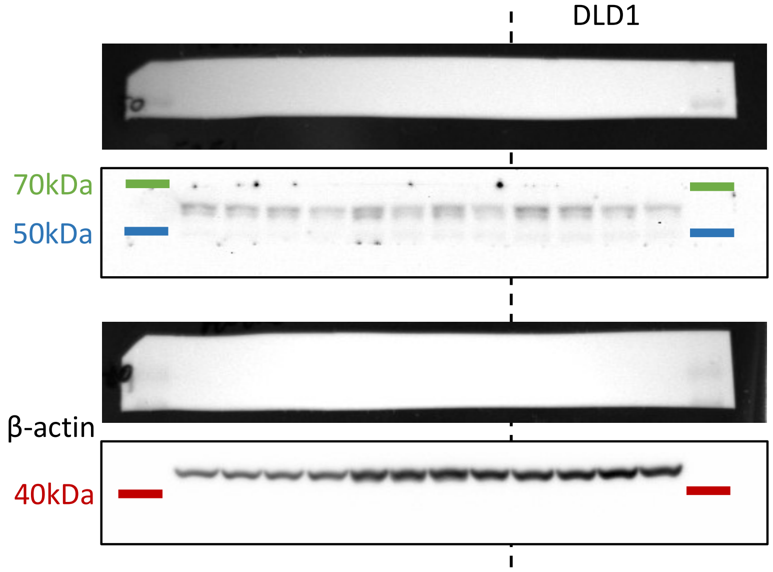

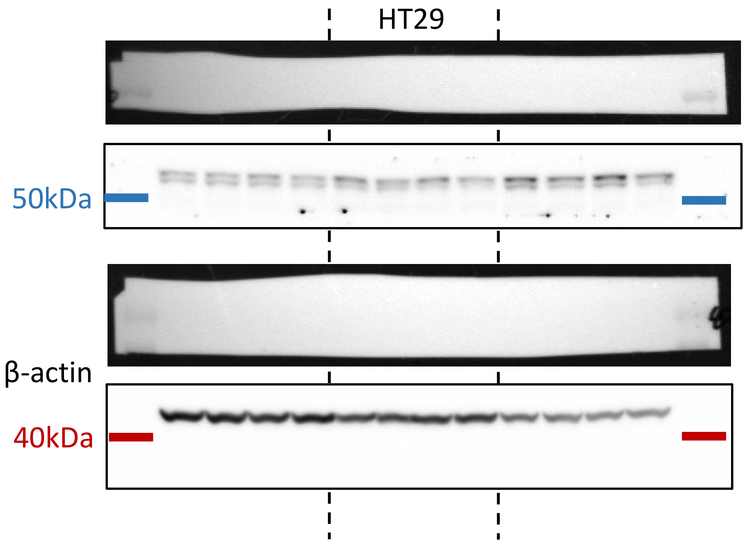

ppRb for Figure 3A, 3C

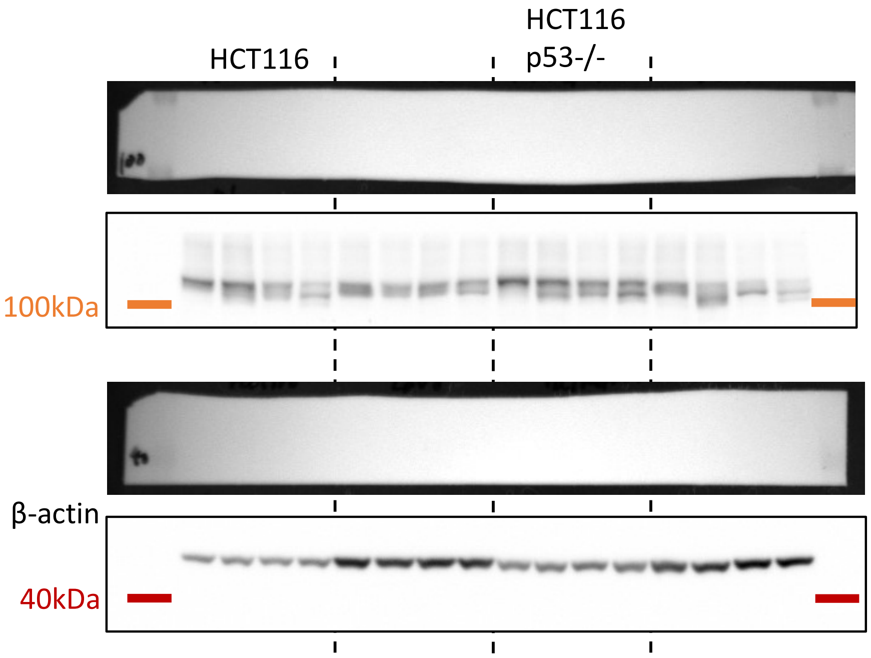

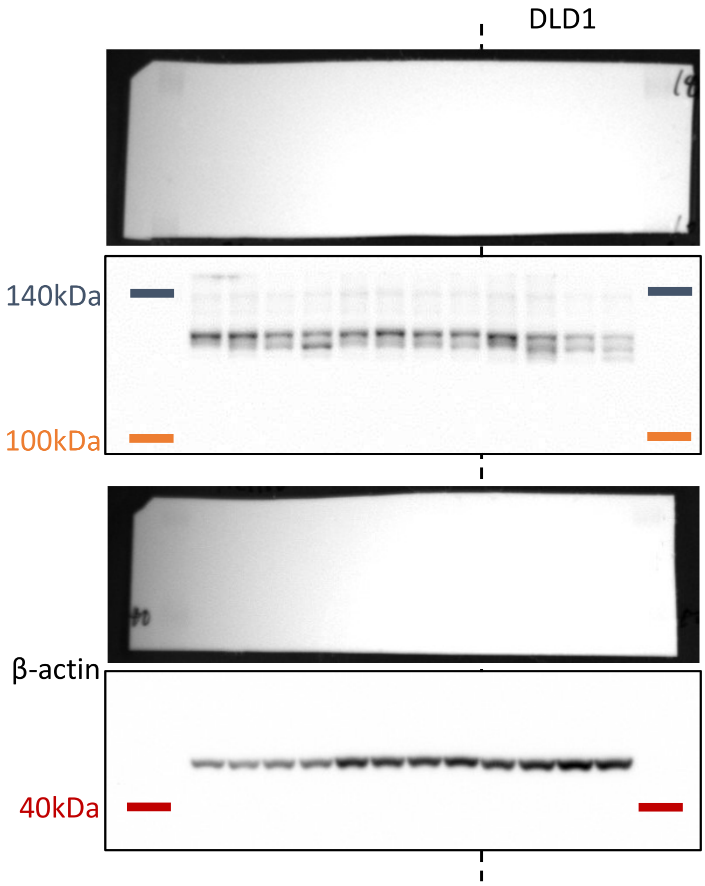

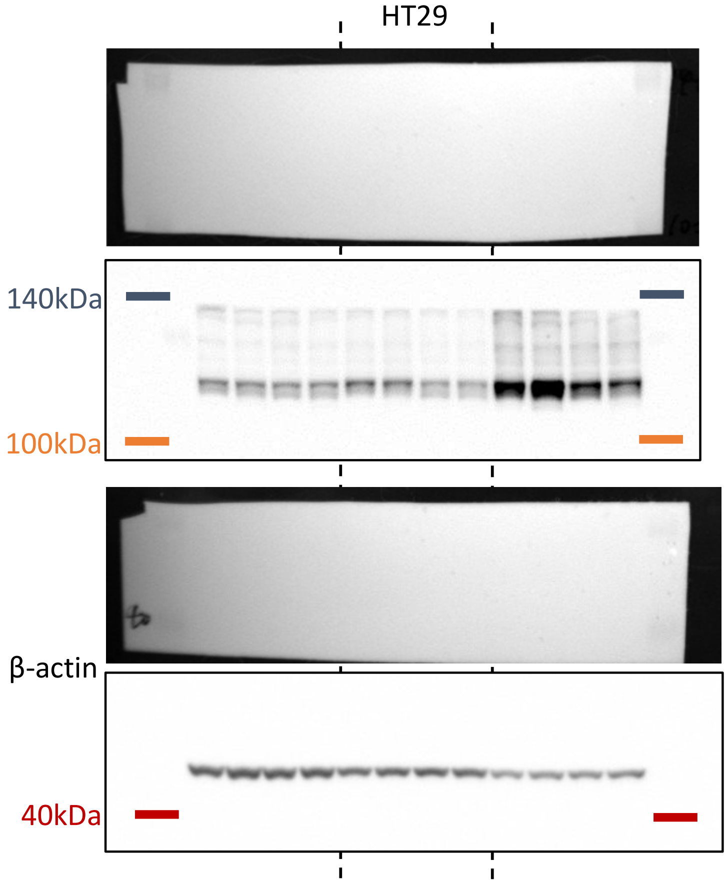

p53 for Figure 3B

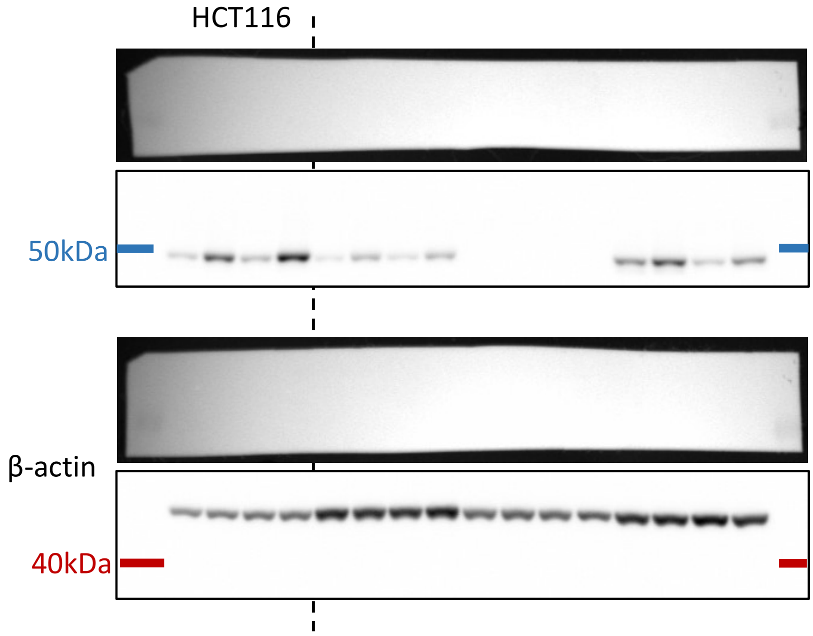

p21 for Figure 3B, 3C

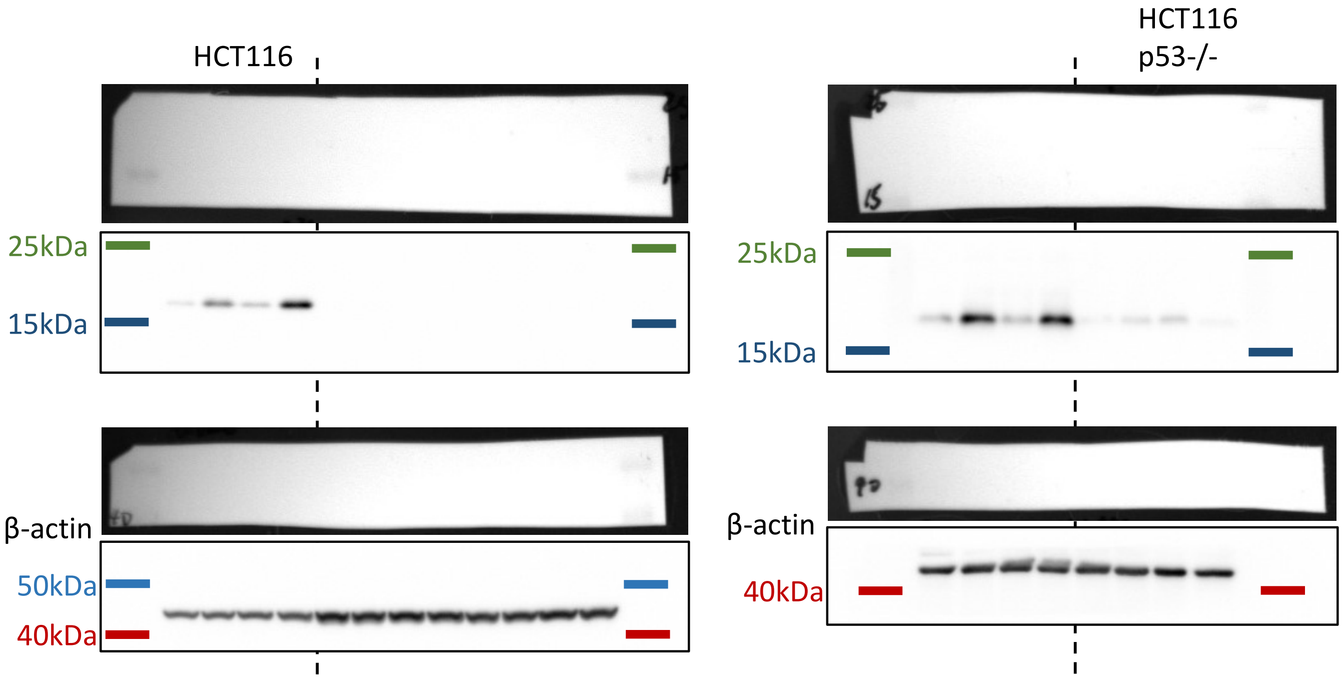

cleaved-PARP for Figure 4E

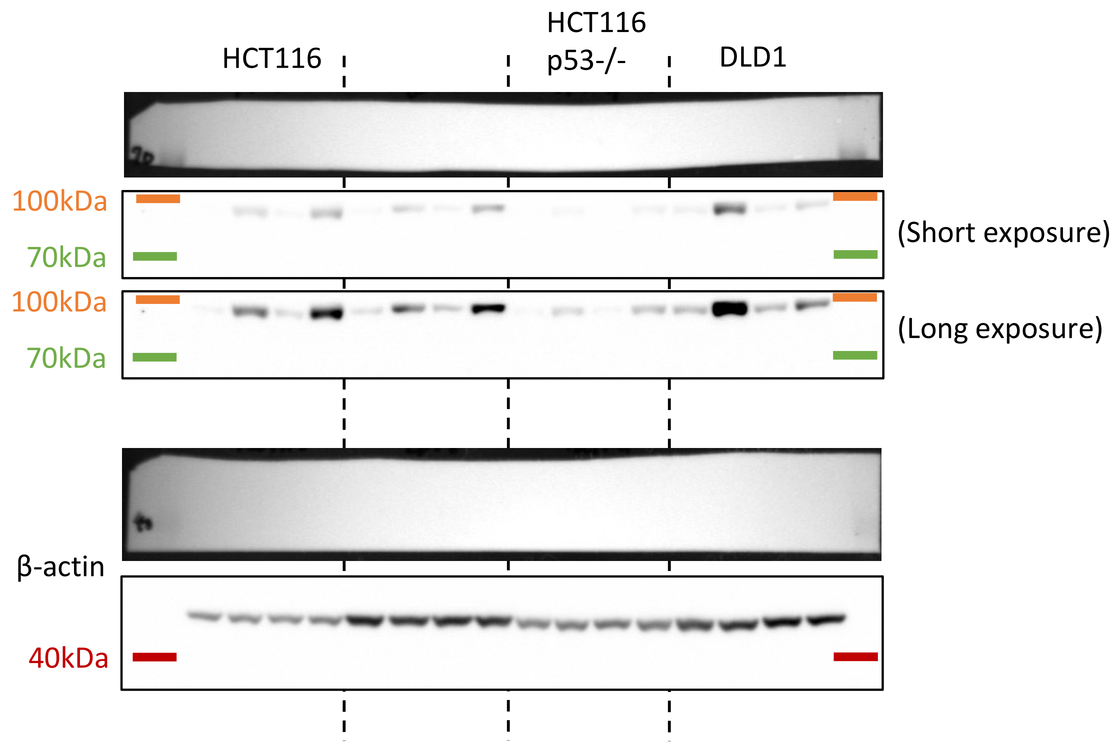

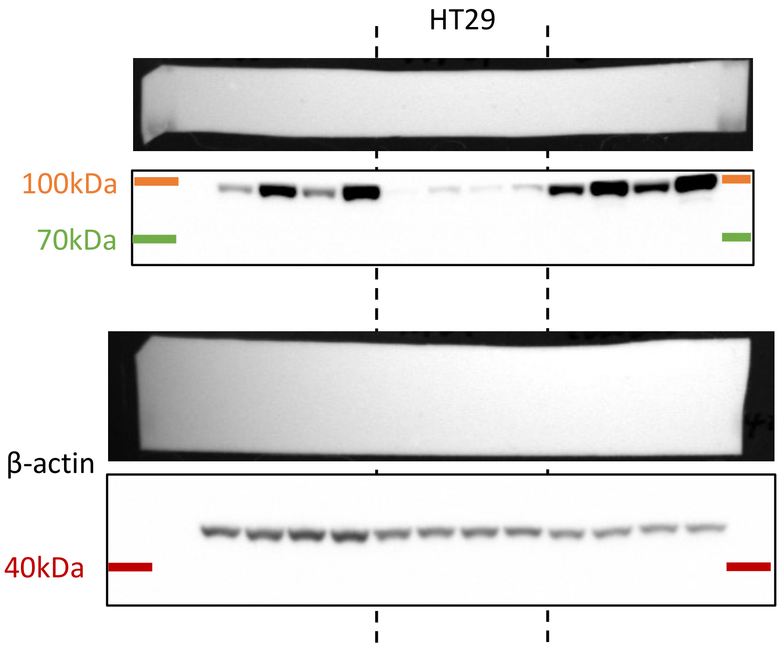

cleaved-Caspase 8, 9 for Figure 4F

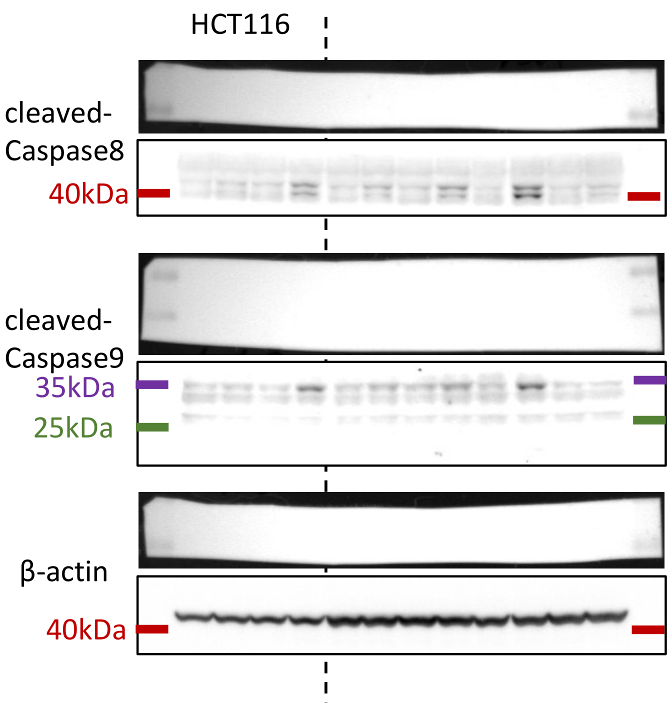

Fas for Figure 4F

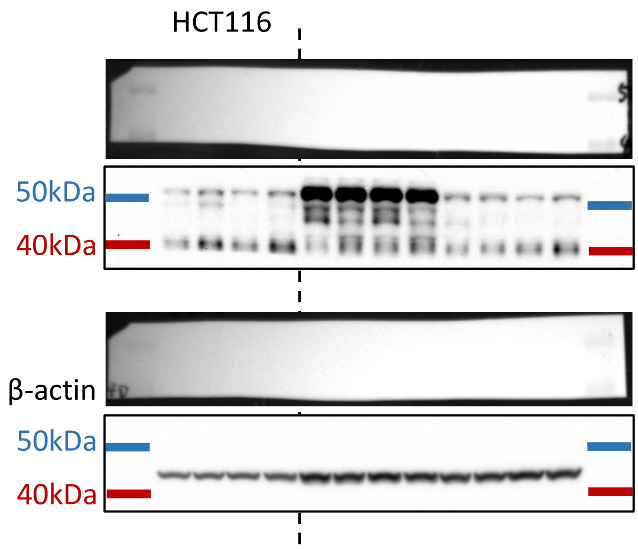

PUMA for Figure 4F

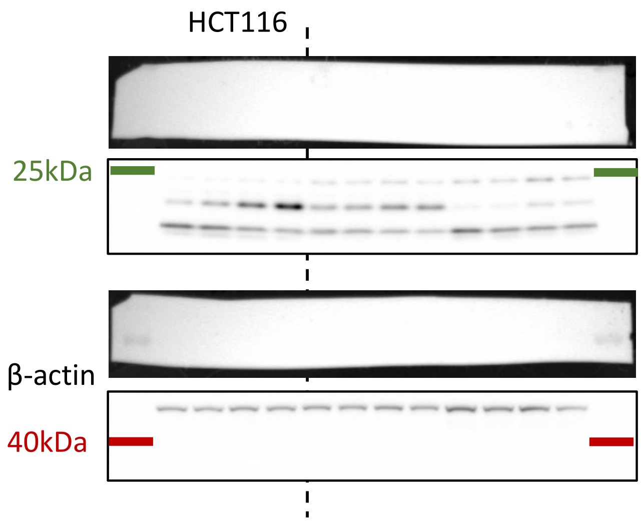

Supplement: Multimedia component 1 [file mmc1.pdf]
