# Supplementary material for: Combination of AURKA inhibitor and MEK inhibitor strongly enhances G1 arrest and induces synergistic antitumor effect on KRAS or BRAF mutant colon cancer cells
Source: Biochem Biophys Rep. 2025 Jun 9;43:102073. doi: 10.1016/j.bbrep.2025.102073 (PMC12180963; doi:10.1016/j.bbrep.2025.102073)
Supplement: Multimedia component 2 [file mmc2.docx]

| **Primary antibody** | | | |
| --- | --- | --- | --- |
| **Antigen** | **Type** | **Catalog number** | **Supplier** |
| β-actin | rabbit polyclonal | PM053 | MBL |
| cleaved-Caspase 8 | rabbit monoclonal | #9496 | Cell Signaling Technology |
| cleaved-Caspase 9 | rabbit polyclonal | #9505 | Cell Signaling Technology |
| cleaved-PARP | rabbit polyclonal | #9541 | Cell Signaling Technology |
| E2F1 | mouse monoclonal | 05-379 | MilliporeSigma |
| FAS | rabbit monoclonal | #4233 | Cell Signaling Technology |
| P21 | mouse monoclonal | #2946 | Cell Signaling Technology |
| P53 | mouse monoclonal | MON7015 | MONOSAN |
| phospho-ERK | mouse polyclonal | AP3906a | Abcepta |
| PUMA | rabbit polyclonal | #4976 | Cell Signaling Technology |
| Rb | mouse monoclonal | 554136 | BD Biosciences |
| **Secondary antibody** | | | |
| **Antigen** | **Type** | **Catalog number** | **Supplier** |
| mouse IgG | HRP-Linked whole Ab from Sheep | NA931 | Cytiva |
| Rabbit IgG | HRP-Linked whole Ab from Donkey | NA934 | Cytiva |

**Table S1.** List of antibodies used in the study.

|  | **IC_50_ Value** | |
| --- | --- | --- |
| **Cell lines** | **MK-5108 (µM)** | **Trametinib (nM)** |
| HCT116 | 0.36 | 9.32 |
| DLD1 | 28.1 | 759 |
| HT29 | 3.21 | 1.20 |
| HCT116p53-/- | 0.25 | 4.79 |

**Table S2.** List of IC₅₀ values of MK-5108 and trametinib for each cell line.


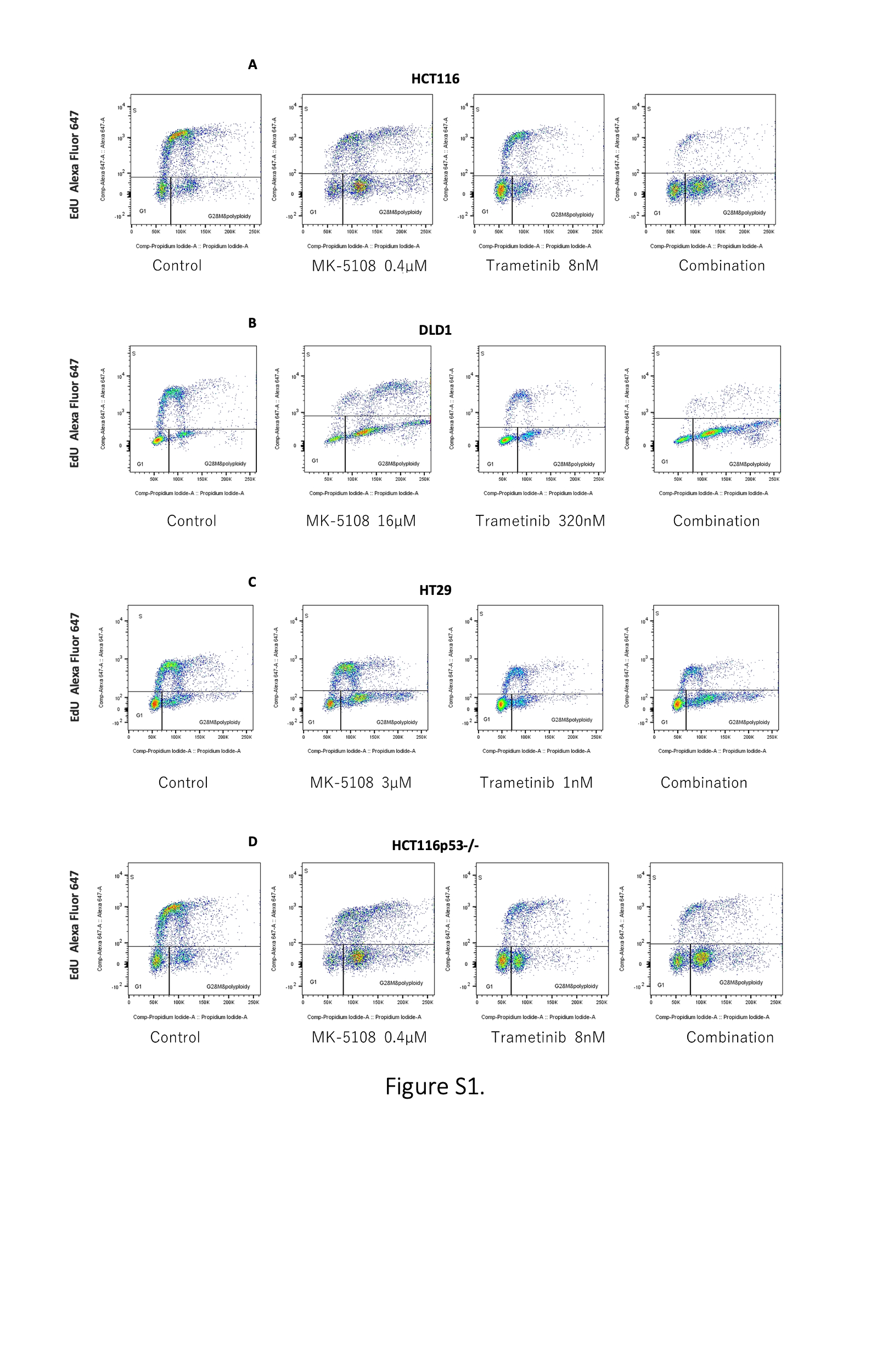
**Figure S1.** Flow cytometry dot plots of EdU assay in all cell lines.


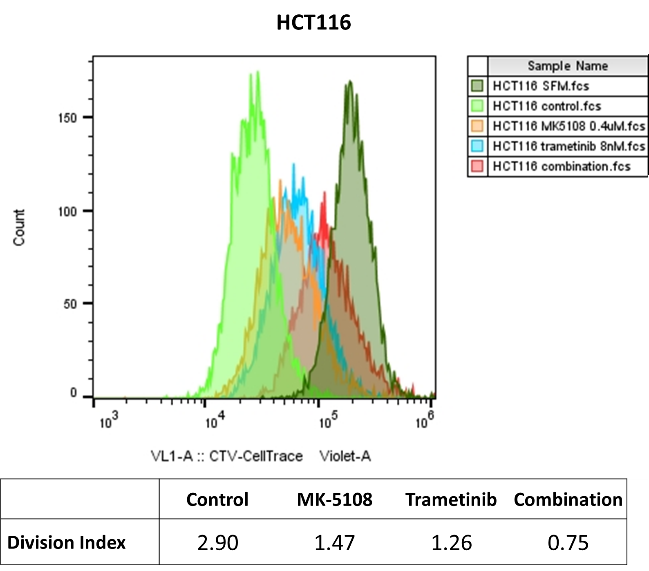


**Figure S2.** Cell proliferation suppression effects of MK-5108 and trametinib in HCT116 cells.

Cells were cultured overnight. Subsequently, cells were stained with CellTrace™ Violet Cell Proliferation Kit (Thermo Fisher). After 3 days of culture, cells were harvested, and flow cytometry was performed using Attune NxT (Thermo Fisher) and analyzed with FlowJo. Cells cultured in serum-free medium were used as non-proliferating cells. The amount of cell proliferation was calculated using the division index.


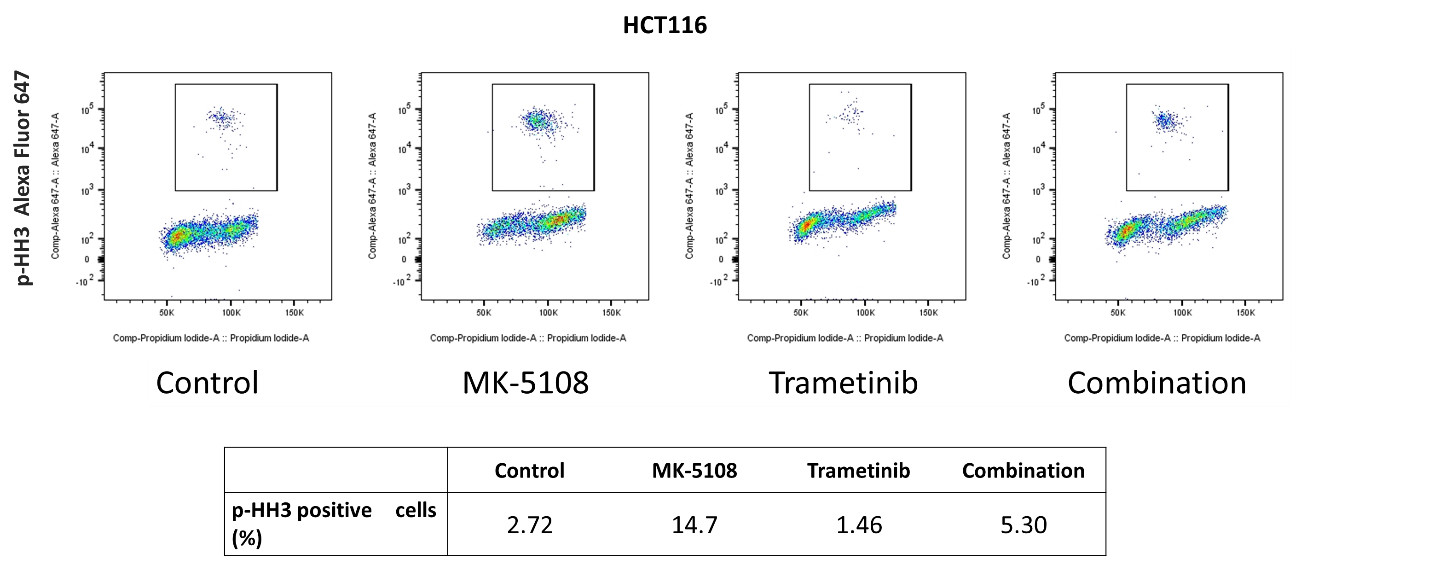


**Figure S3.** Flow cytometry dot plots of HCT116 cells.

Cells were exposed to MK-5108 and/or trametinib for 24 hours and analyzed by flow cytometry. p-HH3 is a marker of mitosis, with positive cells indicating the M phase. Anti-p-HH3 antibody was purchased from BioLegend (San Diego, CA, US).


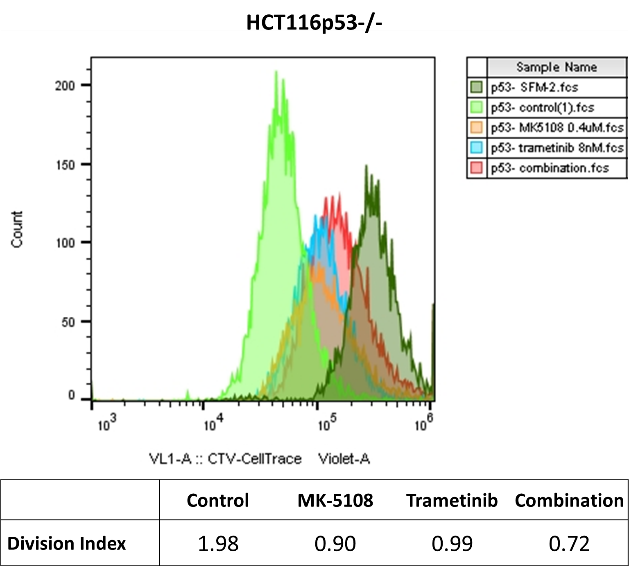


**Figure S4.** Cell proliferation suppression effects of MK-5108 and trametinib in HCT116p53-/- cells.

Cells were exposed to drugs for 3 days and analyzed by flow cytometry using CellTrace violet.


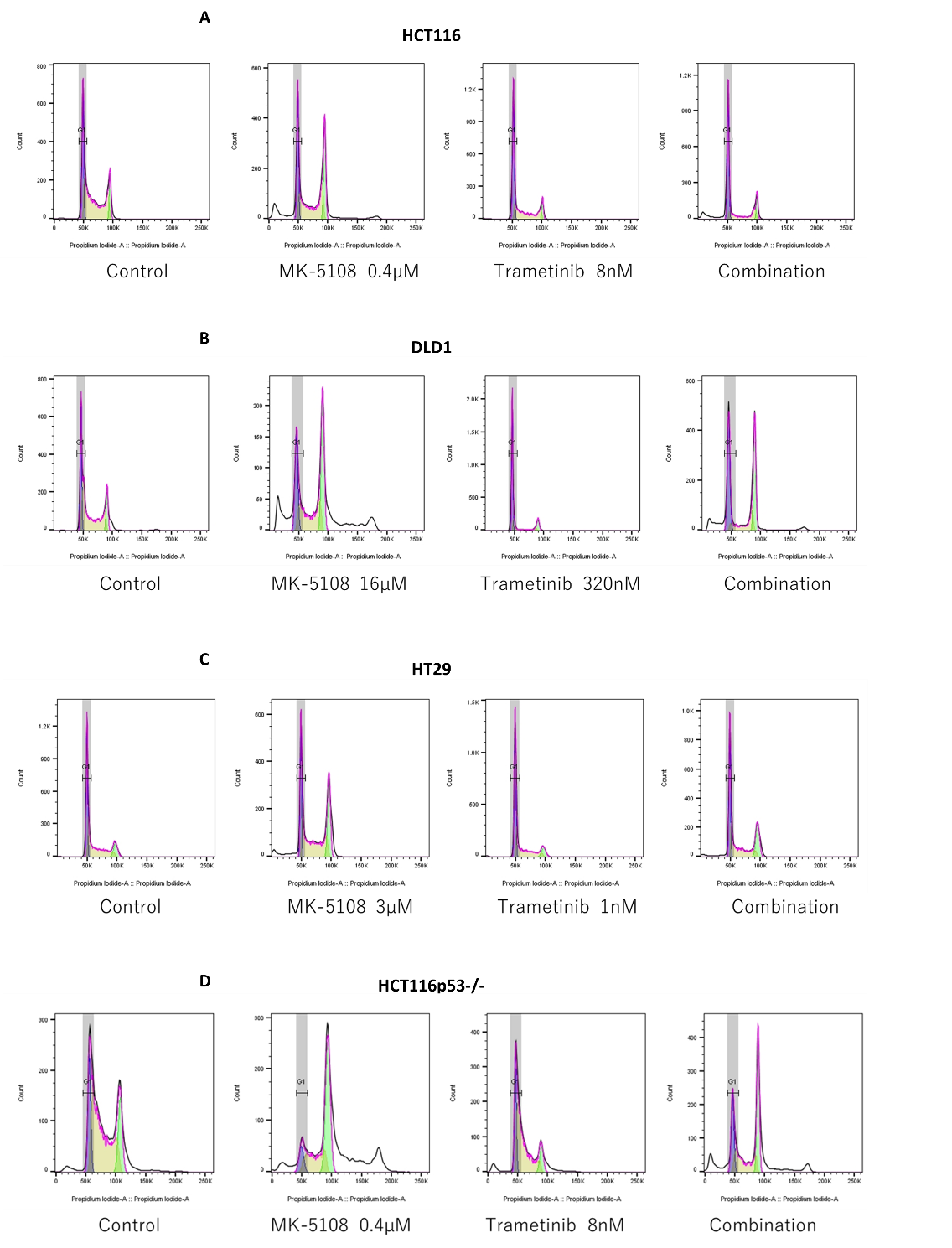


**Figure S5.** Histogram of PI-stained cells analyzed by flow cytometry using FlowJo software.


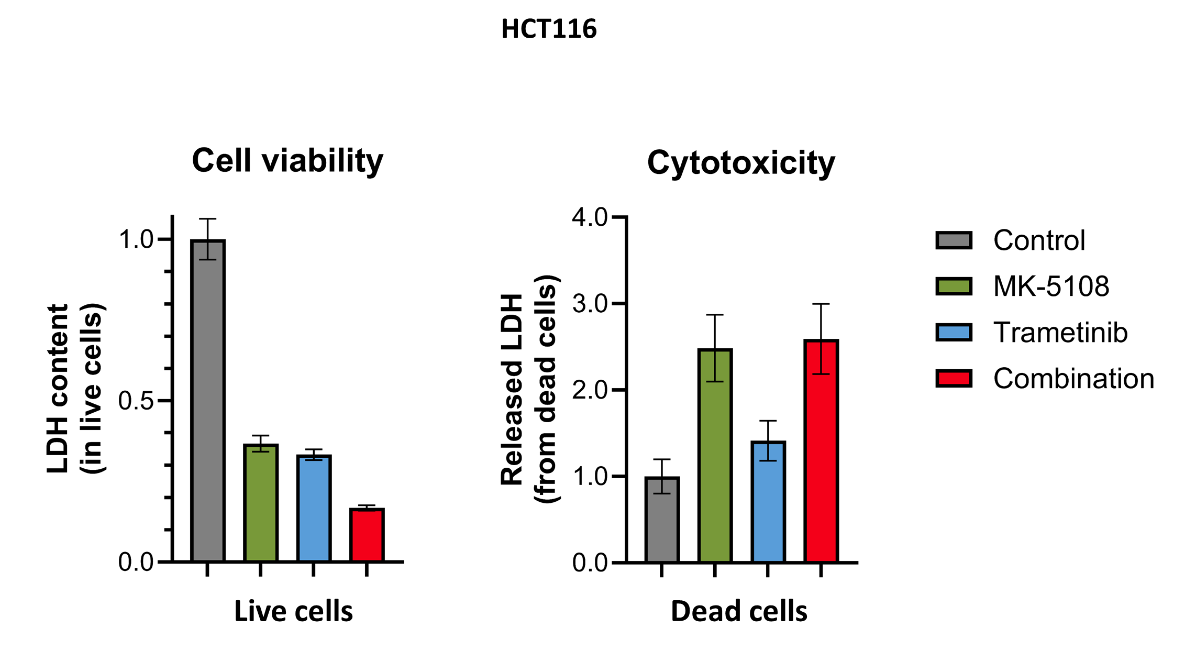


**Figure S6.** Cytotoxicity of MK-5108 and trametinib in HCT116 cells.

Cells were exposed to drugs for 3 days and cytotoxicity was determined by LDH assay. The left panel shows LDH contents contained in cells, indicating cell viability. The right panel shows LDH contents in medium, released from dead cells and indicating cytotoxicity.


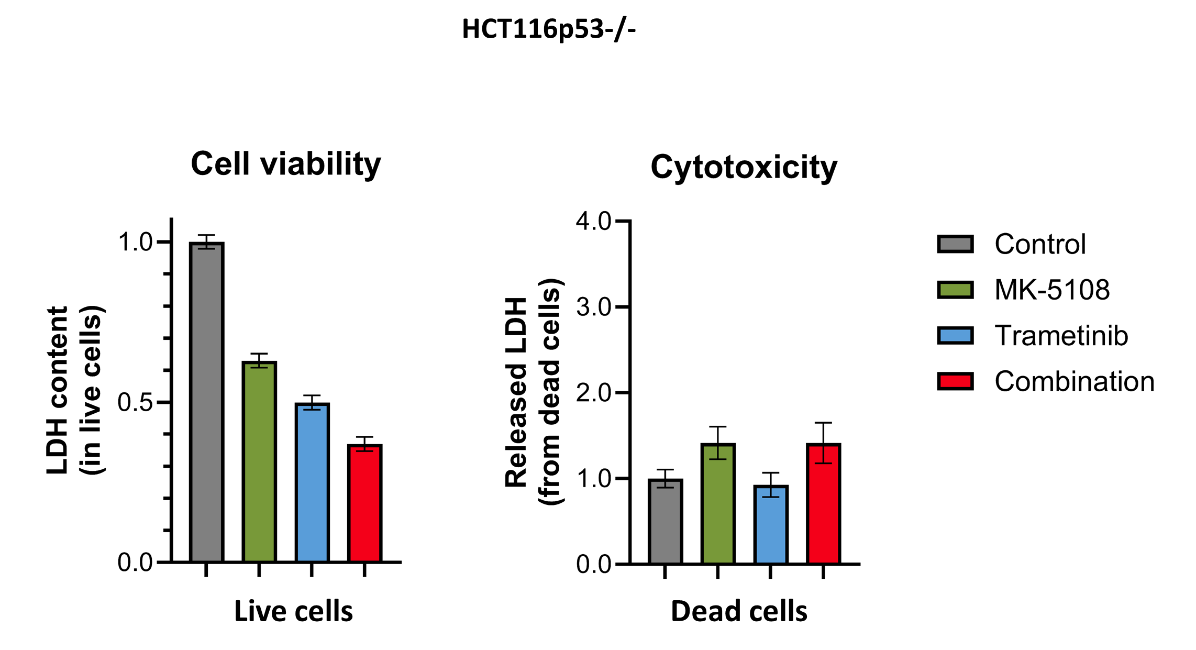


**Figure S7.** Cytotoxicity of MK-5108 and trametinib in HCT116p53-/- cells.
